# *Ex-Vivo* Human-Sized Organ Machine Perfusion: A Systematic Review on the Added Value of Medical Imaging for Organ Condition Assessment

**DOI:** 10.3389/ti.2024.12827

**Published:** 2024-09-04

**Authors:** Jan L. Van Der Hoek, Marleen E. Krommendijk, Srirang Manohar, Jutta Arens, Erik Groot Jebbink

**Affiliations:** ^1^ Multi-Modality Medical Imaging Group, TechMed Centre, University of Twente, Enschede, Netherlands; ^2^ Engineering Organ Support Technologies Group, Department of Biomechanical Engineering, University of Twente, Enschede, Netherlands

**Keywords:** machine perfusion, heart, kidney, liver, organ condition, medical imaging, human, large animal

## Abstract

Machine perfused *ex-vivo* organs offer an excellent experimental platform, e.g., for studying organ physiology and for conducting pre-clinical trials for drug delivery. One main challenge in machine perfusion is the accurate assessment of organ condition. Assessment is often performed using viability markers, i.e., lactate concentrations and blood gas analysis. Nonetheless, existing markers for condition assessment can be inconclusive, and novel assessment methods remain of interest. Over the last decades, several imaging modalities have given unique insights into the assessment of organ condition. A systematic review was conducted according to accepted guidelines to evaluate these medical imaging methods, focussed on literature that use machine perfused human-sized organs, that determine organ condition with medical imaging. A total of 18 out of 1,465 studies were included that reported organ condition results in perfused hearts, kidneys, and livers, using both conventional viability markers and medical imaging. Laser speckle imaging, ultrasound, computed tomography, and magnetic resonance imaging were used to identify local ischemic regions and quantify intra-organ perfusion. A detailed investigation of metabolic activity was achieved using ^31^P magnetic resonance imaging and near-infrared spectroscopy. The current review shows that medical imaging is a powerful tool to assess organ condition.

## Graphical Abstract

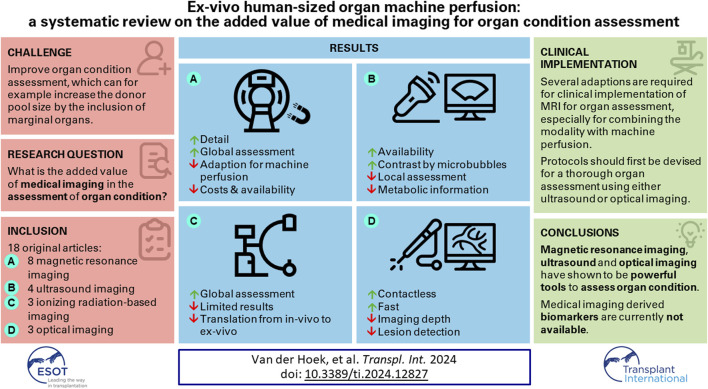

## Introduction

*Ex-vivo* machine perfused organs are a widely used model for experimental research, for example, on drug delivery and transplantation [1, 2]. By (re)perfusing organs *ex-vivo*, an environment can be created that allows for the study of organs in a simulated *in vivo* situation. One reason why *ex-vivo* machine perfused organs are chosen over *in vitro* phantoms is the biological complexity, which is difficult to replicate in the latter. Moreover, the experimental flexibility that *ex-vivo* machine perfused organs offer is generally not attainable in *in vivo* research in technical, operational, and ethical sense. While the technique has already been used and improved for over 150 years, interest has spiked in recent decades due to an increase in clinical relevance and the desire to reduce animal experiments [3, 4]. The main challenge in the research field is to increase the preservation time of organs, which requires a better understanding of the preservation parameters and a more reliable assessment of organ condition [5, 6].

Preservation parameters have been studied extensively, however, the influence of these parameters on organ condition is complex and not fully understood, which is further confounded by the variations in the used perfusion systems, like system components, perfusate types, and hemodynamics [7]. If the organ condition is assessed in these studies, it is often done by measuring viability markers such as perfusate lactate levels, oxygen consumption or organ-specific parameters like bile production in the liver. Often, multiple markers are used, and results are combined to offer information on the condition of the organ, but reliability of existing markers remains questionable [8]. There is, thus, no consensus on the usage of methods to assess organ condition [9].

Novel non-invasive biomarkers are needed to increase reliability of organ condition assessment, to improve and standardize the use of *ex-vivo* perfused organs in research. Several promising biomarkers have been reported that derive from the field of medical imaging [10–12]. These markers could give unique insights into organ condition assessment on both a macroscopic as well as a microscopic scale. Medical imaging can for example, be used to investigate tissue perfusion for the early identification of ischemic regions, but a direct metabolic assessment has also been reported [13, 14].

The goal of this systematic literature review is to give an overview of the added value of medical imaging in the assessment of organ condition in *ex-vivo* perfusion setups. Various imaging biomarkers that have been used for assessment of human-sized machine perfused hearts, kidneys, and livers will be described and compared.

## Materials and Methods

The literature review was conducted using the preferred reporting items for systematic review and metaanalyses (PRISMA) guidelines [15].

### Search Strategy

Two authors (J.H. and M.K.) conducted the literature search independently after reaching consensus on a search query. The same query was used in three databases: Scopus (Elsevier, Amsterdam, Netherlands), PubMed (National Center for Biotechnology Information, Bethesda, United States), and Web of Science (Clarivate Analytics PLC, London, UK) on April 3rd, 2023. The following search headings were used to find eligible studies: (“isolated” OR “*ex vivo*” OR “*ex situ*”) AND (“organ” OR “kidney” OR “liver” OR “heart”) AND (“perfus*” OR “machine perfus*” OR “NMP”) AND (“MRI” OR “magnetic resonance imaging” OR “photoacoustic*” OR “computed tomography” OR “ultrasound” OR “medical imaging” OR “diagnostic imaging”). A resurgence in the use of machine perfusion took place in the early 2000s with the arrival of hollow fibre oxygenators, which increases the possibilities and accuracy for organ condition assessment [4, 16]. Therefore, papers published between 2000 and 2023 were included to ensure coverage of relevant articles after this resurgence. Duplicates were removed using EndNote (version 20.3, Clarivate, London, UK) based on their title, authors, the year of publication, and the journal of publication. Articles were then imported in Rayyan Intelligent Systematic Reviewer (Rayyan Systems Inc., Cambridge, Massachusetts, United States) for title and abstract screening [17].

### Selection Criteria

Studies were included for full-text assessment based on the following criteria: articles that reported (i) isolated organ *ex-vivo* perfusion results using whole (ii) kidneys, hearts, and livers from (iii) pigs, cows, sheep, dogs, and humans, where during perfusion (iv) condition was assessed by histology, blood gas parameters or organ specific viability markers, and where (v) any form of medical imaging, was applied. Studies that used hypothermic machine perfusion were excluded, as the reduced metabolic activity of hypothermically perfused organs makes the setting less suitable for organ condition assessment [4, 18]. Studies that did not present viability results or that did not report ischemia times were excluded, as the undisclosed status of the perfused organ restricts the comparison and evaluation of the different imaging modalities and methods. Studies that only used medical imaging for therapeutic reasons were excluded for the same reason. Case studies and reviews were excluded. Disagreements were discussed and resolved by consulting a third author (E.G.J.).

### Data Extraction

The full texts were reviewed independently by J.H and M.K and a final selection was made by comparing the data extractions. Publication details, including the authors and year of publication, were collected. Animal and organ characteristics including type and weight were extracted, together with information on the condition of the organs based on the assessment by both conventional biomarkers as well as medical imaging modalities.

## Results

### Literature Search

**FIGURE 1:**
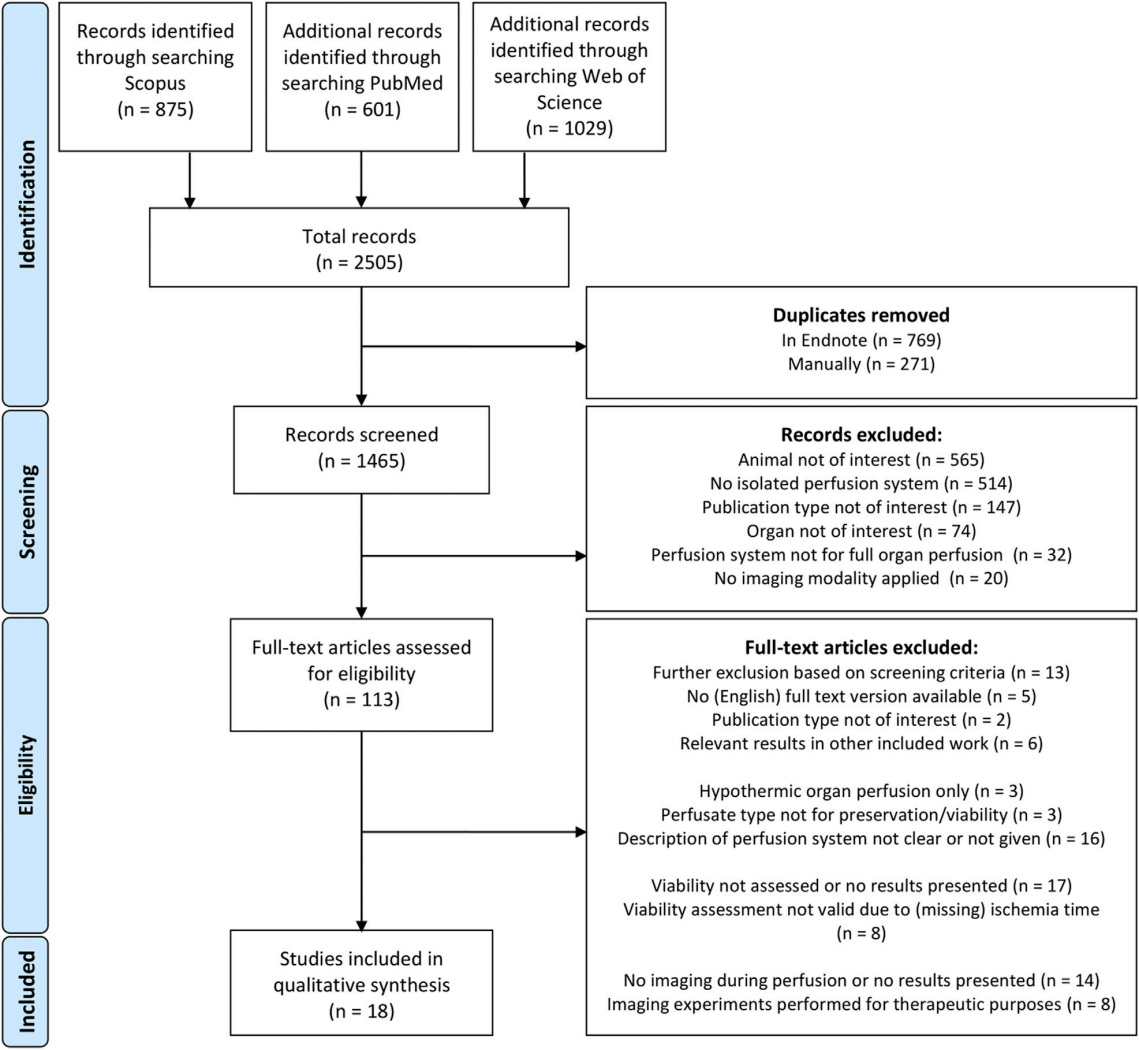
PRISMA flow diagram.

A total of 2,505 abstracts were identified. Figure 1 depicts the flow chart of the study selection, which resulted in 18 included studies. These studies are grouped according to the used imaging modalities, which includes 8 studies with magnetic resonance imaging (MRI) [10, 11, 19–24], 4 studies with ultrasound imaging [13, 25–27], 3 studies with ionizing radiation based imaging [28–30] and 3 studies with optical based imaging [12, 14, 31].

### Study Characteristics

An overview of the study designs and hemodynamics is given in Table 1. The variation in machine perfusion parameters that was mentioned in the introduction is highlighted by the variation in hemodynamics. Additional details can be found in the Supplementary Material for all tables in this review.

**TABLE 1:** Study design and input parameters.

Publication details	Study design					Hemodynamics	
Authors	Animal of study	Number of organs	Organ type and size	Perfusion time		Flow	Pressure
Agius 2022 [19]	Pigs: 45 kg	32	Kidney		4 h	-	40 mmHg systolic 20 mmHg diastolic
Alzaraa 2013 [25]	Pigs: 45–60 kg	10	Liver 1,659 ± 372 g		6 h	Hepatic artery: 210–300 mL/min Portal vein: 730–1,100 mL/min	Hepatic artery: 83–89 mmHg Portal vein: 22–28 mmHg
Bendjelid 2003 [28]	Pigs: 20–25 kg	15	Heart		1.5 h	1 mL/g/min, constant	3 mmHg preload 70 mmHg afterload
Fodor 2022 [12]	Human: BMI avg. 26	21	Liver		Average 20 h (17-27 h)	Hepatic artery: >150 mL/min Portal vein: >500 mL/min	-
Heeman 2021 [14]	Pigs: 130 kg	6	Kidney 338.1 ± 24 g		4 h	Sinusoidal, 60 BPM 150–200 mL/min	85 mmHg
Izamis 2014 [13]	Pigs	22	Liver 1,200-1800 g		3 h	Hepatic artery: 200–300 mL/min Portal vein: 600–900 mL/min	Hepatic artery: 100–112 cm H_2_O Portal vein: <10 cm H_2_O
Liu 2007 [20]	Pigs: 25–30 kg	3	Liver		-	-	-
Mariager 2020 [11]		4	Kidney 131 ± 5 g		2 h	170 mL/min	-
Mariager 2021 [21]	Pigs: 40 ± 3 kg	3	Heart 277 ± 11 g		2 h	300–400 mL/min	75–85 mmHg
Pelgrim 2017 [29]	Pigs: 110 kg	10	Heart		-	Non-stenotic: 1,400 mL/min	Non-stenotic: 54 mmHg
Ribeiro 2020 [26]	Pigs: 40 ± 4 kg	17	Heart		4 h	Working mode 1.8 L/min/m^2^	Langendorff: 40 mmHg Working mode: 25–30 mmHg (diastole)
Schutter 2021 [22]	Pigs: 130 kg Human	Pigs: 9 Human: 4	Kidney Pigs: 291 ± 42 g Human: 224 ± 69 g		4.5 h	Pig: mean 83–129 mL/min/100g Human: mean 143–244 mL/min/100g	85 mmHg
Schutter 2023 [10]	Pigs: 130 kg or 40 ± 2 kg Human	Pigs: 26 Human: 4	Kidney Pigs: 291 ± 42 g or 131 ± 5 g Human 224 ± 69 g		3–4.5 h	Pig: 83–129 mL/min/100g, or 170 mL/min Human: mean 143–244 mL/min/100g	85 mmHg
Singh 2018 [31]	Pigs	8	Liver ±2,170 g		30 min	Hepatic artery: 150 mL/min Portal vein: 150 mL/min	-
Thompson 2020 [27]	Human	10 (5 pairs)	Kidney 199 g–331 g		7 h	50–120 mL/min/100g	Mean arterial pressure of 75 mmHg
Vaillant 2016 [23]	Pigs: 40 kg Sheep: 65 kg	Pigs: 20 Sheep: 1	Heart		±100 min	Working mode 1,000-1,100 mL/min (unloaded RV) 750-900 mL/min (loaded RV)	Langendorff: 60 mmHg Working mode: 60–70 mmHg (LV afterload)
Valenzuela 2023 [30]	Pigs: 87.40 ± 8 kg	32 (16 pairs)	Kidney		3 h	1 L/min for both kidneys	Peak systolic pressure of 120 mmHg
Yang 2008 [24]	Pigs: 25–35 kg	37	Heart		±80 min	1.5 mL/g/min	70–90 mmHg (LV systole) 0–5 mmHg (LV diastole)

### Study Assessment

#### Conventional Organ Condition Assessment

When evaluating medical imaging for organ condition assessment, several conventional biomarkers were reported in the included literature for reference. These conventional biomarkers can be categorized in four groups: hemodynamics, histology, blood (gas) parameters, and organ specific parameters. The biomarker overview is given in the context of medical imaging method evaluation, a thorough analysis of conventional biomarkers can be found elsewhere [9, 32, 33].

Hemodynamic parameters are measured in 17 of the included studies. The flow, pressure, resistance, and temperature can be analysed based on their trends after organ reperfusion. Resistance should decrease after reperfusion, where a deviation can indicate organ damage or oedema formation [14, 29]. Hemodynamic-based assessment can become more elaborate in beating heart models by for example, a contractility assessment [26].

Histological assessment with biopsies is used in 10 studies for a microscopic analysis of organ tissue. Biopsies are often taken at multiple locations to approach a more global evaluation, and most studies also take biopsies at multiple time instances to analyse the effect of reperfusion. Histological assessment gives mostly qualitative information on a cellular level, however, these results are also quantified in some studies, expressed by injury scores [19]. Biopsies were analysed for organ specific structures, like glomerular integrity and tubular dilatation in kidney cortical biopsies [19], and sinusoid and hepatocyte structures in liver biopsies [13]. Several types of staining were also used that are organ agnostic, like periodic acid-schiff staining [22] and hematoxylin and eosin staining [24, 25].

A variety of blood gas parameters are reported in 16 of the included studies. Primarily oxygen (consumption), lactate, glucose, and pH were measured in these studies, which can indicate perfusion deficits and the onset of ischemia, e.g., with a built-up of lactate. Decision-making for liver transplantation is often based on such blood gas parameters, for example, pH stability (7.3–7.45) and the lactate concentration (≤18 mg/dL) for the transplantation of human livers as described by Fodor et al. [12].

Some parameters can also be used that are specific to the organ used in the study, which is described in 17 of the included studies. These include the bile production of the liver and urine production of a kidney, which can be evaluated based on the production quantity and its composition (e.g., pH). In beating heart perfusion studies, the heart beat is generally monitored over the duration of perfusion, where a stable sinus rhythm and ejection fraction are indicative of a healthy heart condition [26, 29].

#### Magnetic Resonance Imaging

A total of 8 studies used MRI during perfusion in the assessment of organs, reporting a wide range of MR techniques (Table 2). A distinction in these techniques can be made based on the detection of abnormalities, e.g., lesions and perfusion defects, or the direct assessment of organ metabolism and function.

**TABLE 2:** Organ assessment with magnetic resonance imaging.

Publication details	Study design		Parameters measured for viability testing				Imaging
Authors	Animal	Organ	Hemodynamics	Histology	Blood parameters/gas contents	Organ-specific	Imaging method
Agius 2022 [19]	Pig	Kidney	-	Cortical biopsies Silver Jones and Periodic AcidSchiff staining	-	Urine output	T2-weighted imaging, dynamic contrast enhanced (DCE) MRI and ^31^P magnetic resonance spectroscopic imaging (pMRSI)
Liu 2007 [20]	Pig	Liver	-	Hemotoxylineosin stained biopsies	Aspartate Aminotransferase (AST)	-	Diffusion weighted MRI with contrast enhancement
Mariager 2020 [11]	Pig	Kidney	Flow, pressure and temperature	-	Blood gas, metabolite and electrolyte values	Urine production	T1 and T2 imaging, hyperpolarized spectroscopy and/or spectral spatial imaging and dynamic contrast enhanced MRI
Mariager 2021 [21]			Flow and pressure	-	Blood gas, metabolite and electrolyte values	-	CINE MR imaging, hyperpolarized spectroscopy and/or spectral spatial imaging and dynamic contrast enhanced MRI
Schutter 2021 [22]	Pig and Human	Kidney	Flow and pressure	Cortical punch biopsies Periodic acid– Schiff staining	Arterial blood gas values	Urine composition and production	Arterial spin labelling (ASL)
Schutter 2023 [10]	Pig and Human	Kidney	Flow, pressure and temperature	-	-	Urine production	T2- and T2*-weighted imaging, arterial spin labelling and hyperpolarized spectroscopy and/or spectral spatial imaging
Vaillant 2016 [23]	Pig and sheep	Heart	Left ventricle pressure and flow	-	pH	Heart rate and action potential	Cine imaging, phase contrast for velocity-encoded cine flow imaging, T1 mapping with late gadolinium enhanced mapping and ^31^P MR spectroscopy.
Yang 2008 [24]	Pig	Heart	Pressure and resistance	Heart slices analysed with near-infrared and various types of staining.	Blood gas values	Heart rate	T1-weighted and contrast (Mn)enhanced MRI

Several studies have shown the capability of MRI to detect lesions and other abnormalities within perfused organs, which would otherwise remain undiscovered. More conventional MR methods are widely used in clinical practice, and several included studies show their suitability for lesion detection. For example, Mariager et al. used a balanced steady state free precession (bSSFP) gradient echo cine imaging sequence on perfused hearts for anatomical assessment, which showed oedema swelling in all hearts [21]. Schutter et al. used a SPACE sequence for T2-weighted anatomical imaging of kidneys to scan for abnormalities [10]. This resulted in the discovery of multiple abnormalities, including a benign cystic structure not visible on the exterior of the kidney, and an ischemic region that was missed by surgical organ inspection. Gadolinium contrast can also be used to highlight lesions by contrast accumulation, which is for example, done with late gadolinium enhanced (LGE) MRI. Vaillant et al. used LGE T1-mapping to identify a myocardial lesion in one heart, which was missed by native T1mapping [23].

Assessment of the entire organ with MRI is also reported in several studies, which can be performed without contrast administration. In a kidney perfusion study by Schutter et al., intrarenal flows were analysed using a method known as arterial spin labelling (ASL) [22]. This method could capture the redistribution mechanism of the kidney, by relative measurements of the cortico-medullar ratio. In the case of heart perfusion, assessment is also possible with MRI based on the functionality of a working heart model with e.g., cine MRI. Hemodynamic values were determined with this method in two studies, which were comparable to *in vivo* values found in literature for the working heart model [23], but different for Langendorff perfused hearts [21].

Administration of contrast is used to better distinguish areas of interest, which is done in dynamic contrast enhanced (DCE) MRI. In a study by Mariager et al., the cortical perfusion in kidneys was measured with this method [11]. The function of the kidney was also assessed with this method by calculating the glomerular filtration rate, which was comparable to values found in literature and could serve as a biomarker. Gadolinium contrast is also used in diffusion weighted MRI, which is predominantly used for diagnosis of acute cerebral ischemia [34]. However, Liu et al. found that the apparent diffusion coefficient (ADC) value derived using diffusion weighted MRI could not discriminate between ischemic and non-ischemic regions in a perfused porcine liver, contradicting *in vivo* result in the same study [20]. In the condition assessment of hearts, Yang et al. introduce manganese as an alternative to gadolinium contrast [24]. Manganese has a fairly rapid uptake in healthy heart cells through the calcium channels, which directly gives a readout of heart condition by serving as a calcium analogue [35]. The sensitivity of manganese enhanced cardiac MRI is highlighted in this study, as ischemic regions were detected that did stain positive for triphenyl tetrazolium chloride, indicating healthy tissue.

The metabolic state of organs can also be analysed directly, by for example, mapping the oxygen consumption in entire organs. Schutter et al. analysed this in perfused kidneys with the functional MR method T2*-mapping, which measures deoxygenated haemoglobin. The T2* signal dropped especially in the functionally important renal cortex when oxygen delivery was halted, thus indicating the onset of ischemia. The myocardial energetic status of perfused hearts was measured by Vaillant et al. with ^31^P MR spectroscopy, using a high-field MR scanner and a coil tuned for both ^1^H and ^31^P [23]. ATP values can be derived by the resulting spectra from this method, which showed that the myocardial energetic status was preserved. Alternatively, energetic status of perfused organs can be assessed by hyperpolarized [1-^13^C]pyruvate MRI. Injected hyperpolarized pyruvate is converted in the tissue into bicarbonate for aerobic metabolism and into lactate for an anaerobic conversion, which can be assessed directly by hyperpolarized spectroscopy in combination with spectral spatial (SPSP) imaging. Schutter et al. used this method to assess perfused kidneys, whereas Mariager et al. used this method to assess both perfused hearts and kidneys in subsequent studies. All studies found a very high lactate conversion rate that was inconsistent with *in vivo* values, which is possibly caused by the altered metabolic profile of *ex-vivo* perfused organs [10, 11, 21].

#### Ultrasound

The assessment of organs was investigated with ultrasound in 4 studies (Table 3). The assessment with ultrasound was mostly based on perfusion, but two other methods have also been reported. For the detection of anatomic abnormalities, conventional B-mode ultrasound imaging was applied by Izamis et al. to scan all 30 included livers, however, no abnormalities were found [13]. The functional analysis of beating hearts was conducted by Ribeiro et al. using surface echocardiography [26]. Significant differences in the left ventricle circumferential strain and right ventricle fractional area change were found in a beating heart donor group and a cardiac death group, which could not be related to post-transplant outcomes.

**TABLE 3:** Organ assessment with ultrasound imaging.

Publication details	Study design		Parameters measured for viability testing				Imaging
Authors	Animal	Organ	Hemodynamics	Histology	Blood parameters/gas contents	Organ-specific	Imaging method
Alzaraa 2013 [25]	Pig	Liver	Flow and pressure	Tru-Cut biopsies Hematoxylineosin staining	Oxygen consumption	Bile production	Contrast Enhanced Ultrasound (CEUS)
Izamis 2014 [13]	Pig	Liver	Flow, pressure and resistance	Hematoxylineosin staining	Oxygen consumption and pH	Bile production	B-mode ultrasound and dynamic contrast enhanced ultrasound (DCEUS)
Ribeiro 2020 [26]	Pig	Heart	Flow, pressure and resistance	-	Blood gas and oxygen consumption	Biventricular function	Surface echocardiography
Thompson 2020 [27]	Human	Kidney	Flow, temperature, pressure and resistance	Histopathological assessment analysis	Blood gas and biochemical	Urine production	Contrast Enhanced Ultrasound (CEUS) MicroFlow Imaging (MFI) and Doppler ultrasound

Assessment of perfusion with ultrasound can be performed with and without a contrast agent. Thompson et al. used MicroFlow imaging Doppler ultrasound for subjective assessment of perfusion. Microflow imaging is a high resolution ultrasound technique that allows for mapping of smaller vessels with more accurate flow measurements in these vessels, compared to conventional ultrasound methods [27, 36]. In the same study, Sonovue (Bracco, Milan, Italy) contrast microbubbles were injected for contrast enhanced ultrasound (CEUS) measurements, which could capture the redistribution mechanism of the kidney. Perfusion was investigated in more detail in two liver perfusion studies, where dynamic contrast enhanced ultrasound (DCEUS) was applied with Sonovue contrast microbubbles to generate time intensity curves. Alzaraa et al. used DCEUS and found a delayed and reduced wash-in in poorly perfused areas and flat curves in non-perfused areas, which were identified as ischemic regions by hematoxylin-eosin staining. Izamis et al. used DCEUS to monitor the perfusion, where microbubbles were injected hourly in both the portal vein and the hepatic artery [13]. Blood clots were identified that were not removed by the initial flush, and the gradual removal of these clots was visualised.

#### Ionizing Radiation Based Imaging Modalities

Only a few ionizing radiation based imaging modalities were found in the included literature (Table 4). Fluoroscopy was performed by Valenzuela et al. in perfused porcine kidney pairs to measure characteristic lengths and angles in the main renal arteries [30]. Pelgrim et al. applied dynamic dual source computed tomography (CT) imaging for blood flow quantification in Langendorff perfused porcine hearts [29]. The mean myocardial blood flow per segment was measured using CT for different stenosis grades of the circumflex artery. A quantitative dynamic CT perfusion analysis could distinguish between ischemic and non-ischemic myocardial segments. A fluorescent microsphere method that is accurate for the quantification of myocardial blood flow was also used and compared to the flow values quantified by CT. While similar trends were visible, the myocardial blood flow was found to be consistently lower when quantified by CT. Bjendelid et al. applied PET for imaging the myocardial status of a perfused porcine heart by monitoring the uptake of injected ^18^F fluorodeoxyglucose (FDG) [28]. This marker for glucose uptake increased linearly over time and a homogeneous distribution was observed over the myocardium. However, FDG could not be correlated with the metabolic increase expected from dobutamine stimulation.

**TABLE 4:** Organ assessment with radiation based imaging modalities.

Publication details	Study design		Parameters measured for viability testing				Imaging
Authors	Animal	Organ	Hemodynamics	Histology	Blood parameters/gas contents	Organ-specific	Imaging method
Bendjelid 2003 [28]	Pig	Heart	Flow and pressure	-	Blood gas values	Heart rate	Positron emission tomography (PET)
Pelgrim 2017 [29]	Pig	Heart	Flow and pressure	Fluorescent microspheres with alcoholic-KOH dissolving	Glucose	Heart rate	Computed tomography (CT)
Valenzuela 2023 [30]	Pigs	Kidney (pairs)	Flow, pressure and temperature	None taken	-	Urine production and composition	Fluoroscopy

#### Optical Imaging Modalities

Optical modalities have also been reported in the assessment of organs, which are mostly focused on the superficial microcirculation (Table 5). Singh et al. used Laser Doppler Flowmetry (LDF) to measure relative changes in the microcirculation in a small field of view (FOV) (<1 mm^2^) [31, 37]. LDF showed that the microcirculation improved after reperfusion. Laser speckle contrast imaging (LSCI) is another optical method and allows relative measurement of the microcirculation in a larger FOV compared to LDF. Heeman et al. compared renal blood flows in a porcine model to normalized laser speckle perfusion units (FOV = 14 × 19 cm) [14]. The onset of ischemia was visible using LSCI, whereas the tissue discoloration due to ischemia took some time. LSCI demonstrated a high correlation with side-stream dark-field (SDF) imaging, which can detect subtle changes in the microcirculation on a red blood cell level [38]. Only a moderately correlation between LSCI and the externally measured renal blood flow was found in a flow ramping experiment, due to the redistribution effect of the kidney. For the analysis of human livers for transplantation, hyperspectral imaging is applied to acquire information on tissue oxygenation, perfusion, and haemoglobin and water concentration [12]. The oxygen saturation, tissue haemoglobin level, and microperfusion increased significantly after the start of perfusion. Livers that were transplanted, according to guidelines for e.g., lactate levels and pH, showed an enhanced microperfusion, measured by the near-infrared perfusion index. Especially the near-infrared perfusion index and the relative distribution of water in the tissue (TWI) show a good correlation with the conventional biomarkers, lactate, and pH respectively.

**TABLE 5:** Organ assessment with optical imaging.

Publication details	Study design		Parameters measured for viability testing				Imaging
Authors	Animal	Organ	Hemodynamics	Histology	Blood parameters/gas contents	Organ-specific	Imaging method
Fodor 2022 [12]	Human	Liver	Flow	-	Blood gas values	Bile production and bile pH	Hyperspectral imaging
Heeman 2021 [14]	Pig	Kidney	Flow, pressure, temperature and resistance	-	-	Urine production	Laser speckle contrast imaging (LSCI) and sidestream dark-field (SDF) imaging
Singh 2018 [31]	Pig	Liver	Temperature	Hematoxylin-eosin staining	pH	Bile production	Laser Doppler Flowmetry (LDF)

## Discussion

A wide variety of imaging modalities have been found in the reviewed literature. Depending on the goal of the viability assessment, several imaging modalities and methods could be considered. The most comprehensive investigation of organ condition can be conducted with magnetic resonance imaging (MRI), which offers a vast range of specific methods for analysis of morphology, perfusion, heart function, and metabolism. The main downsides of MRI are its costs and availability, which is especially problematic in clinical practice for monitoring organs for extended duration. Ultrasound imaging can also be used for analysis of morphology, perfusion, and heart function, where especially the introduction of contrast microbubbles showed promising results for the analysis of perfusion. While ultrasound is a relatively inexpensive imaging option that is more widely available, full organ assessment is limited both by the field of view as well as the reduced anatomical information that ultrasound can provide compared to MRI. Ionizing radiation-based techniques showed that perfusion analysis can only be performed qualitatively, where the reported method for metabolic assessment was limited in the *ex-vivo* setting. The optical based modalities showed promising results for the analysis of both perfusion and metabolism, although these are limited by the penetration depth.

### Lesion Detection

For the detection of lesions, multiple MRI sequences have been reported in the included literature. Next to the more conventional T1 and T2 weighted imaging, late gadolinium enhanced (LGE) imaging with contrast gadolinium was also applied, which could identify lesions missed by native T1-weighted imaging [23]. Multiple studies have shown that LGE is a suitable method for the detection of cardiac lesions, like cardiomyopathies, ischemic heart disease, and fibrosis [39, 40]. However, LGE is limited for diseases that are spread out over the heart, like diffuse fibrosis [41].

While no lesions were detected with ultrasound in the included studies, the imaging modality has shown its capability to detect lesions of specific nature, like cystic structures [42]. Moreover, the introduction of contrast microbubbles with contrast enhanced ultrasound (CEUS) has shown that benign and malignant lesions can be identified and distinguished [43].

Contrary to the findings in this review, multiple *in vivo* studies have shown that computed tomography (CT) has a high sensitivity for lesion detection [42]. MRI and CT have shown comparable overall sensitivity for lesion detection, with specific strengths for each modality (e.g., CT for calcified lesions and MRI for tumours smaller than 20 mm in diameter) [44, 45]. While ultrasound is more widely available, it is often only used for initial screening for lesions, due to a relatively lower sensitivity for lesion detection compared to MRI and CT.

### Perfusion Analysis

Dynamic contrast enhanced (DCE) MRI is the most used method for the perfusion analysis with MRI in the reviewed literature, which showed adequate perfusion in all studies. DCE MRI is mostly used with gadolinium contrast, but manganese is another contrast option that is mostly used in the assessment of hearts. However, several studies have also addressed a downside of manganese contrast, which is induced cardiovascular dysfunction [46, 47]. MRI ASL is a non-invasive alternative to the invasive DCE MRI, showing a moderate to high correlation in a comparison study [48]. Interestingly, the non-invasive diffusion-weighted MRI, which is widely used in clinic for the detection of brain ischemia and tumours, did not translate to the *ex-vivo* situation in a study by Liu et al., although this pilot study based this conclusion on only one blood-perfused liver [20, 34].

For the perfusion analysis of organs, CEUS was mostly used to monitor the tissue perfusion over the duration of the experiment. While the method shows that small details like the removal of blood clots after reperfusion and the onset of ischemia can be mapped, the method does require contrast microbubble injections for each measurement. Micro-flow imaging is an alternative ultrasound method that does not require contrast enhancement. This method shows a higher sensitivity to detecting the vascularity of hepatocellular carcinoma compared to other non-contrast enhanced ultrasound methods, albeit lower than the sensitivity found with CEUS [49].

Perfusion was analysed with CT in one included study, which also indicated that perfusion analysis can only be done qualitatively as CT underestimated the myocardial blood flow [29]. Other studies do describe the quantitative evaluation of tissue perfusion with perfusion CT, which is for example, used in the diagnosis of abdominal cancer [50].

Superficial perfusion can be analysed in great detail by optical modalities like side-stream dark field (SDF) imaging and laser doppler flowmetry (LDF), although these modalities are very limited by their field of view for the analysis of entire organs. Laser speckle imaging and hyperspectral imaging could both analyse the microcirculation of perfused organs on a larger scale, which were also good indicators for transplantation outcomes as described by Fodor et al. [12].

### Metabolic Analysis

In the metabolic assessment of organs, T2*-weighted imaging can be used to measure the tissue oxygenation. The T2* signal changed significantly when the oxygen supply to a perfused kidney was interrupted, although the authors mention that this method cannot be used to quantify organ dysfunction at this moment [10]. The method is used *in vivo* in the metabolic analysis of transplanted kidneys, specifically for the detection of early allograft dysfunction [51]. A metabolic analysis of perfused hearts was performed with ^31^P MR spectroscopy, which showed comparable values to *in vivo* studies for healthy hearts. Hyperpolarized spectroscopy and spectral spatial (SPSP) imaging provided insight into the metabolic distribution over perfused organs, however, the high lactate values that were found in all three studies compared to *in vivo* results from literature suggest results should be interpreted with caution. Considering the hypotheses that Mariager et al. describe in both the heart perfusion and the kidney perfusion studies, the technique requires *ex-vivo* validation to develop it as a biomarker in this setting.

The metabolic analysis with ^18^F fluorodeoxyglucose (FDG) positron emission tomography (PET) imaging did not correlate with the expected metabolic increase that was induced in perfused hearts [28]. A large retrospective study by Sprinz et al. analysed FDG uptake in the liver, lungs and brain, where only in the brain FDG was a significant marker for glucose uptake [52].

Hyperspectral imaging showed its ability to assess human liver condition prior to transplantation in a proof-ofconcept study [12]. However, the superficial relative blood oxygenation StO_2_ could not discriminate between transplanted and non-transplanted livers, whereas the near-infrared perfusion index and the relative distribution of water over the organ could be used to this end.

### Functional (Cardiac) Analysis

Cine cardiac imaging was used for the functional assessment of both Langendorff-perfused hearts, as well as hearts in working mode. Results showed that a complete replication of all parameters of the *in vivo* situation is difficult. Both included studies that performed the functional assessment found a decreased left ventricular ejection fraction and aortic flow compared to *in vivo* data with cardiac MRI. However, for the functional assessment of hearts, studies have indicated that this might be sufficient as only specific parameters are important in the functional assessment of hearts for e.g., transplantation outcomes [26, 53].

Echocardiography could not be used to predict post-transplant outcomes [26]. In this study as well, the *ex-vivo* working heart setting was difficult to replicate and the poor echocardiography post-transplant outcome correlation was ascribed to the evaluation method where only a steady pre-load was used in the assessment.

### Clinical Implementation

This review has shown that several medical imaging derived parameters correlate with established biomarkers, while also providing detailed and locoregional information on the condition of organs that current biomarkers cannot provide. However, several adaptations are required for clinical implementation of medical imaging based organ assessment, particularly for pre-transplant organ assessment.

The most comprehensive analysis of organ condition is provided by MRI, where several methods provided detailed feedback on the condition of the entire perfused organ. However, the modality does also require significant adaptions in order to be implemented in the current workflow. Firstly, the Faraday cage limits placement of the machine perfusion system to the control room. For perfusion of an organ in the MRI, special MRI-compatible organ chambers should be used, as well as elongated tubing to connect the organ across the Faraday cage. The latter has a severe impact on the heat loss through the tubing and the damping of flow pulsatility over the tubing, requiring major adaptions to the workflow. Secondly, usage of the MRI prior to transplantation may not be feasible in all institutions. While studies have shown that machine perfusion can preserve a heart for over 10 h for successful transplantation [54], this timeframe together with the limited availability and priorities within MRI scheduling might impede the adoption of MRI for organ assessment.

Ultrasound is a more flexible alternative concerning costs, availability and repeated measurements over the preservation time, where especially contrast microbubbles offer detailed perfusion information. Contrast microbubbles have shown an excellent safety profile in patients [55], and the *ex-vivo* machine perfusion setting also facilitates non-invasive administration of microbubbles. Nevertheless, ultrasound is a 2D modality that in general provides less information than 3D MRI. To enhance ultrasound assessment, protocols should be devised for the assessment of entire organs. Alternatively, 3D ultrasound might be an option for such an assessment, although accurate real-time image reconstruction remains challenging [56].

The optical imaging method hyperspectral imaging could be a suitable option for organ assessment, where the study by Fodor et al. found that hyperspectral imaging derived parameters aligned with viability markers commonly used during liver normothermic perfusion [57]. The contactless method can offer real-time information on both perfusion and the metabolic state of the organ, and permanent integration with the perfusion setup is the most straightforward compared to the other modalities. The main limitation of optical based organ assessment is the penetration depth, which limits the extent of organ assessment to the surface. Moreover, lesions are poorly detected by optical modalities, where a combination with ultrasound might be a solution.

### Limitations

In this review, the imaging methods and modalities were grouped and compared on several functionalities, e.g., perfusion analysis and lesions detection. A large variation in these studies was found for the used perfusion system characteristics and parameters, where standardization is necessary. The heterogeneity of the studies complicates the comparison of the imaging methods and results, requiring cautious interpretation.

### Conclusion

Magnetic resonance imaging (MRI) offers a wide range of methods for accurate assessment of organ condition, where especially functional MRI offers unique insights. Ultrasound is a more flexible alternative that becomes especially significant with the introduction of contrast microbubbles. The results for computed tomography (CT) and other ionizing radiation based imaging modalities are limited for *ex-vivo* machine perfused organs, however, literature does show its potential in the organ condition assessment. While optical modalities are slightly more experimental compared to the other modalities, analysis of the superficial microperfusion has shown to be a quick and good method to assess organ condition. Although a detailed overview of the different imaging methods for organ condition analysis could be given, results were not conclusive on the suitability of medical imaging features as biomarkers to report on organ condition.

## Data Availability

The original contributions presented in the study are included in the article/Supplementary Material, further inquiries can be directed to the corresponding author.

## References

[1] Stevens LJ Zhu AZX Chothe PP Chowdhury SK Donkers JM Vaes WHJ Evaluation of Normothermic Machine Perfusion of Porcine Livers as a Novel Preclinical Model to Predict Biliary Clearance and Transporter-Mediated Drug-Drug Interactions Using Statins. Drug Metab Disposition (2021) 49(9):780–9. 10.1124/DMD.121.000521 34330719

[2] Valenza F Rosso L Coppola S Froio S Palleschi A Tosi D *Ex vivo* LUNG Perfusion to Improve Donor LUNG Function and Increase the Number of Organs Available for Transplantation. Transpl Int (2014) 27(6):553–61. 10.1111/tri.12295 24628890 PMC4241040

[3] Wolfgang B Frank M Heinz Hermann W. History of Extracorporeal Circulation: The Conceptional and Developmental Period. J Extra Corpor Technol. (2003) 35(3):172–83. 10.1051/ject/2003353172 14653416

[4] Jing L Yao L Zhao M Peng LP Liu M. Organ Preservation: FROM the PAST to the Future. Acta Pharmacologica Sinica (2018) 39(5):845–57. 10.1038/aps.2017.182 29565040 PMC5943901

[5] Matsuno N Kobayashi E. Challenges in Machine Perfusion Preservation for Liver Grafts FROM Donation After Circulatory Death. Transpl Res (2013) 2(19):19. 10.1186/2047-1440-2-19 PMC3896750 24283383

[6] Li J Lu H Zhang J Li Y Zhao Q. Comprehensive Approach to Assessment of Liver Viability During Normothermic Machine Perfusion. J Clin Translational Hepatol (2023) 11(2):466–79. 10.14218/JCTH.2022.00130 PMC9817053 36643041

[7] Bouari S Eryigit Ö de Bruin RWF Ijzermans JNM Minnee RC, “Optimizing Porcine Donor Kidney Preservation WITH Normothermic or Hypothermic Machine Perfusion: A Systematic Review,” Artif Organs (2021) 45(11):1308–16. 10.1111/aor.14039 34309868 PMC8596691

[8] Panconesi R Flores Carvalho M Mueller M Meierhofer D Dutkowski P Muiesan P Viability Assessment in Liver Transplantation—What Is the Impact of Dynamic Organ Preservation? Biomedicines (2021) 9(2):161–25. 10.3390/biomedicines9020161 33562406 PMC7915925

[9] Brüggenwirth IMA de Meijer VE Porte RJ Martins PN. Viability Criteria Assessment During Liver Machine Perfusion. Nat Biotechnol (2020) 38(11):1260–2. 10.1038/s41587-020-0720-z 33106683

[10] Schutter R van Varsseveld OC Lantinga VA Pool MBF Hamelink TH Potze JH Magnetic Resonance Imaging During WARM Ex VIVO Kidney Perfusion. Artif Organs (2023) 47(1):105–16. 10.1111/aor.14391 35996889 PMC10086841

[11] Mariager CØ Hansen ESS Bech SK Munk A Kjaergaard U Lyhne MD Graft Assessment of the Ex Vivo Perfused Porcine Kidney Using Hyperpolarized [1-13C]pyruvate. Magn Reson Med (2020) 84(5):2645–55. 10.1002/mrm.28363 32557782

[12] Fodor M Lanser L Hofmann J Otarashvili G Pühringer M Cardini B Hyperspectral Imaging as a TOOL for Viability Assessment During Normothermic Machine Perfusion of Human Livers: A Proof of Concept Pilot Study. Transpl Int (2022) 35:10355. 10.3389/ti.2022.10355 35651880 PMC9150258

[13] Izamis ML Efstathiades A Keravnou C Leen EL Averkiou MA. Dynamic ContrastEnhanced Ultrasound of Slaughterhouse Porcine Livers in Machine Perfusion. Ultrasound Med Biol (2014) 40(9):2217–30. 10.1016/j.ultrasmedbio.2014.03.031 25023101

[14] Heeman W Maassen H Calon J van Goor H Leuvenink H van Dam GM Real-Time Visualization of Renal Microperfusion Using Laser Speckle Contrast Imaging. J Biomed Opt (2021) 26(05):056004. 10.1117/1.jbo.26.5.056004 34024055 PMC8140613

[15] Moher D Shamseer L Clarke M Ghersi D Liberati A Petticrew M Preferred Reporting Items for Systematic Review and Meta-Analysis Protocols (PRISMA-P) 2015 Statement. Revista Espanola de Nutricion Humana y Dietetica (2016) 20(2):1–160. 10.1186/2046-4053-4-1 PMC4320440 25554246

[16] Lewandowski K. Extracorporeal Membrane Oxygenation for Severe Acute Respiratory Failure. Crit Care (2000) 4:156–68. 10.1186/cc689 11094500 PMC137254

[17] Ouzzani M Hammady H Fedorowicz Z Elmagarmid A. Rayyan-A Web and Mobile App for Systematic Reviews. Syst Rev (2016) 5(1):210. 10.1186/s13643-016-0384-4 27919275 PMC5139140

[18] Jakubauskas M Jakubauskiene L Leber B Strupas K Stiegler P Schemmer P. Machine Perfusion in Liver Transplantation: A Systematic Review and Meta-Analysis. Visc Med (2022) 38(4):243–54. 10.1159/000519788 36160822 PMC9421699

[19] Agius T Songeon J Klauser A Allagnat F Longchamp G Ruttimann R Subnormothermic *Ex VIVO* Porcine Kidney Perfusion Improves Energy Metabolism: Analysis Using 31P Magnetic Resonance Spectroscopic Imaging. Transpl Direct (2022) 8(10):E1354. 10.1097/TXD.0000000000001354 PMC9514833 36176724

[20] Liu Q Monbaliu D Vekemans K Peeters R De Keyzer F Dresselaers T Can Apparent Diffusion Coefficient Discriminate Ischemic FROM Nonischemic Livers? A Pilot Experimental Study. Transpl Proc (2007) 39(8):2643–6. 10.1016/j.transproceed.2007.08.003 17954198

[21] Mariager CØ Hansen ESS Bech SK Eiskjaer H Nielsen PF Ringgaard S Development of a Human Heart-Sized Perfusion System for Metabolic Imaging Studies Using Hyperpolarized [1-13C]pyruvate MRI. Magn Reson Med (2021) 85(6):3510–21. 10.1002/mrm.28639 33368597

[22] Schutter R Lantinga VA Hamelink TL Pool MBF van Varsseveld OC Potze JH Magnetic Resonance Imaging Assessment of Renal FLOW Distribution Patterns During Ex VIVO Normothermic Machine Perfusion in Porcine and Human Kidneys. Transpl Int (2021) 34(9):1643–55. 10.1111/tri.13991 34448269 PMC9290094

[23] Vaillant F Magat J Bour P Naulin J Benoist D Loyer V Magnetic Resonance-Compatible Model of Isolated Working Heart FROM Large Animal for Multimodal Assessment of Cardiac Function, Electrophysiology, and Metabolism. Am J Physiol Heart Circ Physiol (2016) 310:H1371–80. 10.1152/ajpheart.00825.2015 26968545

[24] Yang Y Gruwel MLW Sun J Gervai P Yang X Kupriyanov VV. Manganeseenhanced MRI of Acute Cardiac Ischemia and Chronic Infarction in Pig Hearts: Kinetic Analysis of Enhancement Development. NMR Biomed (2009) 22(2):165–73. 10.1002/nbm.1297 18756440

[25] Alzaraa A Al-Leswas D Chung WY Gravante G Bruno M West K Contrast-Enhanced Ultrasound Detects Perfusion Defects in an Ex VIVO Porcine Liver Model: A Useful TOOL for the Study of Hepatic Reperfusion. J Artif Organs (2013) 16(4):475–82. 10.1007/s10047-013-0717-1 23813223

[26] Ribeiro RVP Alvarez JS Yu F Adamson MB Paradiso E Mbadjeu Hondjeu AR Comparing Donor Heart Assessment Strategies During *Ex Situ* Heart Perfusion to Better Estimate Posttransplant Cardiac Function. Transplantation (2020) 104(9):1890–8. 10.1097/TP.0000000000003374 32826843

[27] Thompson ER Bates L Ibrahim IK Sewpaul A Stenberg B McNeill A Novel Delivery of Cellular Therapy to Reduce Ischemia Reperfusion Injury in Kidney Transplantation. Am J Transplant (2021) 21(4):1402–14. 10.1111/ajt.16100 32506663

[28] Bendjelid K Canet E Rayan E Casali C Revel D Janier M. ROLE of Glycolysis in the Energy Production for the Non-Mechanical Myocardial WORK in Isolated Pig Hearts. Curr Med Res Opin (2003) 19(1):51–8. 10.1185/030079902125001281 12661781

[29] Pelgrim GJ Das M van Tuijl S van Assen M Prinzen FW Stijnen M Validation of Myocardial Perfusion Quantification by Dynamic CT in an Exvivo Porcine Heart Model. Int J Cardiovasc Imaging (2017) 33(11):1821–30. 10.1007/s10554-017-1171-6 28536897 PMC5682851

[30] Valenzuela TF Schinstock E Kohnle S Latib A Bliagos D Tunev S Preclinical Research Performed on Reanimated/Perfused Swine Kidneys: The Visible Kidney^TM^ Methodologies. Physiol Rep (2023) 11(5):e15630. 10.14814/phy2.15630 36878878 PMC9988650

[31] Singh S Siriwardana P Johnston EW Watkins J Bandula S Illing R Perivascular Extension of Microwave Ablation ZONE: Demonstrated Using an Ex VIVO Porcine Perfusion Liver Model. Int J Hyperthermia (2018) 34(7):1114–20. 10.1080/02656736.2017.1400119 29096566

[32] Guzzi F Knight SR Ploeg RJ Hunter JP. A Systematic Review to Identify Whether Perfusate Biomarkers Produced During Hypothermic Machine Perfusion Can Predict Graft Outcomes in Kidney Transplantation. Transpl Int (2020) 33(6):590–602. 10.1111/tri.13593 32031281

[33] Qian X Shah P Agbor-Enoh S. Noninvasive Biomarkers in Heart Transplant: 2020-2021 YEAR in Review. Curr Opin Organ Transpl (2022) 27(1):7–14. 10.1097/MOT.0000000000000945 PMC8711631 34939959

[34] Baliyan V Das CJ Sharma R Gupta AK. Diffusion Weighted Imaging: Technique and Applications. World J Radiol (2016) 8(9):785–98. 10.4329/wjr.v8.i9.785 27721941 PMC5039674

[35] Wendland MF. Applications of Manganese-Enhanced Magnetic Resonance Imaging (MEMRI) to Imaging of the Heart. NMR Biomed (2004) 17(8):581–94. 10.1002/nbm.943 15761947

[36] Aghabaglou F Ainechi A Abramson H Curry E Kaovasia TP Kamal S Ultrasound Monitoring of Microcirculation: An Original Study FROM the Laboratory Bench to the Clinic. Microcirculation (2022) 29(6–7):e12770. 10.1111/micc.12770 35611457 PMC9786257

[37] Guven G Dijkstra A Kuijper TM Trommel N van Baar ME Topeli A Comparison of Laser Speckle Contrast Imaging WITH Laser Doppler Perfusion Imaging for Tissue Perfusion Measurement. Microcirculation (2023) 30(1):e12795. 10.1111/micc.12795 36524297 PMC10078364

[38] Goedhart PT Khalilzada M Bezemer R Merza J Ince C. Sidestream DARK Field (SDF) Imaging: A Novel Stroboscopic LED Ringbased Imaging Modality for Clinical Assessment of the Microcirculation. Opt Express (2007) 15(23):15101–14. 10.1364/oe.15.015101 19550794

[39] Aquaro GD De Gori C Faggioni L Parisella ML Cioni D Lencioni R Diagnostic and Prognostic ROLE of LATE Gadolinium Enhancement in Cardiomyopathies. Eur Heart J (2023) 25:C130–C136. 10.1093/eurheartjsupp/suad015 PMC10132607 37125322

[40] Vöhringer M Mahrholdt H Yilmaz A Sechtem U. Significance of LATE Gadolinium Enhancement in Cardiovascular Magnetic Resonance Imaging (CMR). Herz (2007) 32(2):129–37. 10.1007/s00059-007-2972-5 17401755

[41] Doltra A Amundsen BH Gebker R Fleck E Kelle S. Emerging Concepts for Myocardial LATE Gadolinium Enhancement MRI. Curr Cardiol Rev (2013) 9:185–90. 10.2174/1573403x113099990030 23909638 PMC3780343

[42] Leão LRDS Mussi TC Yamauchi FI Baroni RH. Common Pitfalls in Renal MASS Evaluation: A Practical Guide. Radiol Bras (2019) 52(4):254–61. 10.1590/0100-3984.2018.0007 31435088 PMC6696749

[43] Jung EM Weber MA Wiesinger I. Contrast-Enhanced Ultrasound Perfusion Imaging of Organs. Radiologe (2021) 61:19–28. 10.1007/s00117-021-00891-7 34378067 PMC8354100

[44] Van Oostenbrugge TJ Fütterer JJ Mulders PFA. Diagnostic Imaging for Solid Renal Tumors: A Pictorial Review. Kidney Cancer (2018) 2(2):79–93. 10.3233/KCA-180028 30740580 PMC6364093

[45] Elbanna KY Kielar AZ. Computed Tomography Versus Magnetic Resonance Imaging for Hepatic Lesion Characterization/Diagnosis. Clin Liver Dis (2021) 17(3):159–64. 10.1002/cld.1089 PMC8043714 33868658

[46] Pan D Schmieder AH Wickline SA Lanza GM. Manganese-Based MRI Contrast Agents: PAST, Present, and Future. Tetrahedron (2011) 67(44):8431–44. 10.1016/j.tet.2011.07.076 22043109 PMC3203535

[47] Jiang Y Zheng W. Cardiovascular Toxicities Upon Manganese Exposure. Cardiovasc Toxicol (2005) 5:345–54. 10.1385/ct:5:4:345 16382172 PMC3980854

[48] Cai W Li F Wang J Du H Wang X Zhang J A Comparison of Arterial SPIN Labeling Perfusion MRI and DCE-MRI in Human Prostate Cancer. NMR Biomed (2014) 27(7):817–25. 10.1002/nbm.3124 24809332

[49] Bae JS Lee JM Jeon SK Jang S. Comparison of Microflow Imaging WITH Color and Power Doppler Imaging for Detecting and Characterizing Blood FLOW Signals in Hepatocellular Carcinoma. Ultrasonography (2019) 39(1):85–93. 10.14366/usg.19033 31759383 PMC6920623

[50] Garbino N Brancato V Salvatore M Cavaliere C. A Systematic Review on the ROLE of the Perfusion Computed Tomography in Abdominal Cancer. Dose-Response (2021) 19(4):15593258211056199. 10.1177/15593258211056199 34880716 PMC8647276

[51] Park SY Kim CK Park BK Kim SJ Lee S Huh W. Assessment of Early Renal Allograft Dysfunction WITH Blood Oxygenation Level-dependent MRI and Diffusion-Weighted Imaging. Eur J Radiol (2014) 83(12):2114–21. 10.1016/j.ejrad.2014.09.017 25452096

[52] Sprinz C Zanon M Altmayer S Watte G Irion K Marchiori E Effect S of Blood Glucose Level on 18F Fluorodeoxyglucose (18F-FDG) Uptake for PET/CT in Normal Organs: An Analysis on 5623 Patients. Sci Rep (2018) 8(1):2126. 10.1038/s41598-018-20529-4 29391555 PMC5794870

[53] Bona M Wyss RK Arnold M Méndez-Carmona N Sanz MN Günsch D Cardiac Graft Assessment in the Era of Machine Perfusion: Current and Future Biomarkers. J Am Heart Assoc (2021) 10(4):e018966–29. 10.1161/JAHA.120.018966 33522248 PMC7955334

[54] Qin G Jernryd V Sjöberg T Steen S Nilsson J. Machine Perfusion for Human Heart Preservation: A Systematic Review. Transpl Int (2022) 35:10258. 10.3389/ti.2022.10258 35401041 PMC8983812

[55] Piscaglia F Bolondi L, Italian Society for Ultrasound in Medicine and Biology SIUMB Study Group on Ultrasound Contrast Agents. The Safety of Sonovue in Abdominal Applications: Retrospective Analysis of 23188 Investigations. Ultrasound Med Biol (2006) 32:1369–75. 10.1016/j.ultrasmedbio.2006.05.031 16965977

[56] Huang Q Zeng Z. A Review on Real-Time 3D Ultrasound Imaging Technology. Biomed Res Int (2017) 2017:6027029. 10.1155/2017/6027029 28459067 PMC5385255

[57] Fodor M Lanser L Hofmann J Otarashvili G Pühringer M Cardini B Hyperspectral Imaging as a TOOL for Viability Assessment During Normothermic Machine Perfusion of Human Livers: A Proof of Concept Pilot Study. Transpl Int (2022) 35:10355. 10.3389/ti.2022.10355 35651880 PMC9150258

